# Effects of Caffeine on Myocardial Blood Flow: A Systematic Review

**DOI:** 10.3390/nu10081083

**Published:** 2018-08-13

**Authors:** Randy van Dijk, Daan Ties, Dirkjan Kuijpers, Pim van der Harst, Matthijs Oudkerk

**Affiliations:** 1 Center for Medical Imaging, University Medical Center Groningen, University of Groningen, 9713 GZ Groningen, The Netherlands; r.van.dijk02@umcg.nl (R.v.D.); d.ties@umcg.nl (D.T.); t.kuijpers@haaglandenmc.nl (D.K.); p.van.der.harst@umcg.nl (P.v.d.H.); 2 Department of Cardiology, University Medical Center Groningen, University of Groningen, 9713 GZ Groningen, The Netherlands; 3 HMC-Bronovo, Haaglanden Medisch Centrum, Department of Radiology, Haaglanden Medisch Centrum-Bronovo, 2597 AX The Hague, The Netherlands

**Keywords:** caffeine, myocardial perfusion, coronary artery disease, adenosine, regadenoson, dipyridamole

## Abstract

Background. Caffeine is one of the most widely consumed stimulants worldwide. It is a well-recognized antagonist of adenosine and a potential cause of false-negative functional measurements during vasodilator myocardial perfusion. The aim of this systematic review is to summarize the evidence regarding the effects of caffeine intake on functional measurements of myocardial perfusion in patients with suspected coronary artery disease. Pubmed, Web of Science, and Embase were searched using a predefined electronic search strategy. Participants—healthy subjects or patients with known or suspected CAD. Comparisons—recent caffeine intake versus no caffeine intake. Outcomes—measurements of functional myocardial perfusion. Study design—observational. Fourteen studies were deemed eligible for this systematic review. There was a wide range of variability in study design with varying imaging modalities, vasodilator agents, serum concentrations of caffeine, and primary outcome measurements. The available data indicate a significant influence of recent caffeine intake on cardiac perfusion measurements during adenosine and dipyridamole induced hyperemia. These effects have the potential to affect the clinical decision making by re-classification to different risk-categories.

## 1. Introduction

Noninvasive and invasive functional measurements are increasingly used to assess myocardial perfusion in both research and the clinical setting. To unmask relevant myocardial perfusion defects, it is essential to achieve maximal hyperemia during these measurements. The most widely used vasodilator agents used to achieve this hyperemic effect are adenosine, regadenoson, and dipyridamole. The hyperemic effect is primarily caused by binding to the adenosine A_2A_-receptor on arteriolar vascular smooth muscle cells [1].

### 1.1. Vasodilator Agent Mechanisms of Action

Both adenosine and regadenoson act by directly binding to adenosine receptors. Adenosine is a nonselective adenosine receptor agonist and binds to all the different adenosine subtypes, including the adenosine A_2B_-receptor subtype [2]. Binding to this receptor causes bronchospasm in patients with hypersensitive airways. Therefore, adenosine cannot be used in patients with either asthma or chronic obstructive pulmonary disease (COPD) [2,3,4]. Regadenoson can safely be used in patients with hypersensitive airways, due to its selective binding to the A_2A_-receptor [5,6,7,8]. Dipyridamole acts as an adenosine re-uptake inhibitor. The inhibited uptake of adenosine by cells increases the extra-cellular adenosine concentration, increasing the amount of adenosine that is available for binding to adenosine receptors.

### 1.2. Caffeine Antagonism

**Figure 1:**
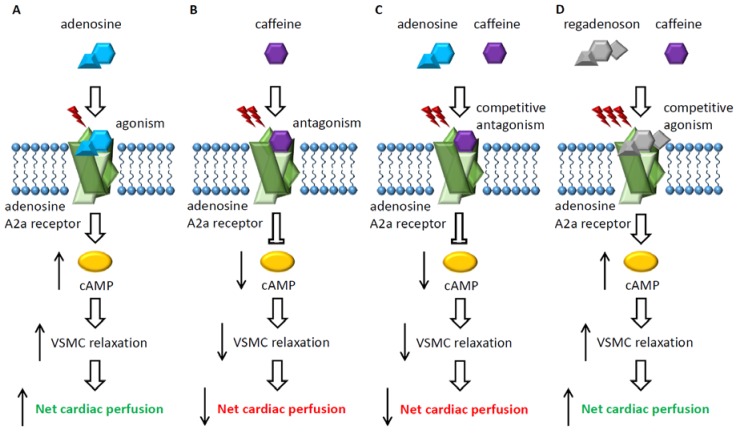
Suggested molecular A_2a_-receptor effects showing adenosine agonism (**A**), caffeine antagonism (**B**), competitive antagonism of caffeine on adenosine (**C**), and competitive agonism of regadenoson on caffeine (**D**).

When adenosine binds to the G-protein coupled A_2a_-receptor, located on cardiac vascular smooth muscle cells, intra-cellular production of cAMP and activation of protein kinase increase, resulting in hyperpolarisation and consequently relaxation of vascular smooth muscle cells. Caffeine is a well-recognized antagonist of adenosine [9]. The competitive antagonistic nature of caffeine for the A_2A_-receptor is a potential cause of achieving insufficient hyperemia, resulting in false-negative functional perfusion measurements [10]. Caffeine limits the binding of adenosine to the receptor and consequently possibly limits cardiac vasodilation and stress adequacy. Regadenoson is a potent selective A_2A_-receptor agonist that is possibly less influenced by caffeine due to the stronger affinity for the receptor. Figure 1 is a simplified graphical illustration of the effect of the vasodilator agents and caffeine on the A_2a_-receptor.

The effects of caffeine on different vasodilator myocardial perfusion measurements remains unclear, and conflicting results have been published. By conducting a review of current literature, two recent debate articles have attempted to shed light on the possible influence of caffeine on myocardial perfusion imaging and its clinical impact [11,12]. However, these papers both fail to provide a complete, unbiased systematic overview of current available evidence on the effects of caffeine on vasodilator myocardial perfusion measurements.

### 1.3. Caffeine Consumption

Caffeine is one of the most widely consumed stimulants worldwide and is present in a wide range of substances such as coffee, soft drinks, energy drinks, tea, and chocolate [13]. The European Food Safety Authority (EFSA) recently published a scientific opinion paper regarding the safety of caffeine [14]. The papers conducted extensive surveys in 22 European countries. They report a wide variability of mean daily caffeine intake per country. The daily intake ranged from 21.8–416.8 mg per day in individuals ≥18 years old, with coffee being the predominant caffeine containing beverage consumed [14]. An average cup of coffee contains approximately 85 mg of caffeine [14]. When taking the average amount of caffeine per cup, the reported coffee intake in the ESFA database roughly translates to a mean coffee intake of 0.25–5 cups. However, it should be recognized that the caffeine dose varies extensively depending on several factors, for example the type of coffee bean and brewing method [15].

### 1.4. Clinical Practice

In clinical practice, patients are generally instructed to refrain from consumption of caffeine containing substances for a period ranging from 12–24 h prior to myocardial perfusion testing. However, the adherence rate of patients to this advice is unclear and serum concentrations of caffeine are not routinely measured in the period preceding the functional measurement. In a study by Banko et al., 36/190 (19%) of patients who screened negative for recent caffeine ingestion by interview still had detectable serum caffeine levels prior to the examination [16]. It is also debatable whether 12 or 24 h caffeine abstinence prior to MPI should be recommended. Carlsson et al., compared coronary flow reserve (CFR) on MRI measured 12 and 24 h after study-induced caffeine ingestion, and showed a significantly lower coronary flow reserve after 12 h caffeine abstinence compared to 24 h caffeine abstinence [17].

### 1.5. Aim of the Study

This systematic review will summarize the evidence regarding the effects of caffeine intake on functional measurements of myocardial perfusion in patients with suspected coronary artery disease (CAD).

## 2. Methods and Results

### 2.1. Protocol and Registration

This systematic review was performed in concordance with the Preferred Reporting Items for Systematic Reviews and Meta-analyses (PRISMA) statement and was registered at PROSPERO under registration number CRD42018092187.

### 2.2. Eligibility Criteria

Participants—healthy subjects or patients with known or suspected CAD. Comparisons—recent caffeine intake versus no caffeine intake. Outcomes—measurements of functional myocardial perfusion. Study design—observational.

### 2.3. Search Strategy

Pubmed, Web of Science, and Embase were searched using a specific electronic search strategy. The following search strategy was used in Pubmed: (“Coffee”[Mesh] OR “Caffeine”[Mesh] OR caffeine [tiab] OR coffee [tiab] OR coffea [tiab]) AND (“Heart” [Mesh] OR “Myocardial Ischemia” [Mesh] OR Myocardi* [tiab] OR Cardiac [tiab] OR cardiovas* [tiab] OR heart [tiab] OR coronar* [tiab]) AND (perfusion* [tiab] OR “Perfusion Imaging” [Mesh] OR “Magnetic Resonance Imaging” [Mesh] OR Magnetic Resonance [tiab] OR Magnetic-resonance [tiab] OR MR [tiab] OR CMR [tiab] OR MRI [tiab] OR “Tomography, X-ray Computed” [Mesh] OR Computed tomograph* [tiab] OR CT [tiab] OR “Positron-Emission Tomography” [Mesh] OR Positron Emission Tomograp* [tiab] OR PET [tiab] OR “Single Photon Emission Computed Tomography Computed Tomography” [Mesh] OR photon Emission Computed Tomograph* [tiab] OR SPECT [tiab] OR “Fractional Flow Reserve, Myocardial” [Mesh] OR “Coronary Angiography” [Mesh] OR Fractional Flow Reserve [tiab] OR FFR [tiab] OR coronary angiograph* [tiab] OR coronary-angiograph* [tiab]). The search strategy for Web of Science and Embase was adjusted according to requirements and preferences of the different databases.

### 2.4. Study Selection and Data Collection

The results from the systematic search of the different databases were collected in Mendeley. Duplicates were removed by using the automatic “check for duplicates” function within Mendeley and an additional manual check for duplicates. Two reviewers independently screened the articles for eligibility using the title and abstract. After title and abstract screening, the results from the reviewers were compared and consensus was achieved in case of discrepancies. The remaining articles were read in full text independently by both reviewers and screened for inclusion. Results of full text screening were compared and discussed afterwards. The data extraction of eligible articles was performed with the use of a predefined template.

### 2.5. Search Results

In total, 702 articles were identified. After duplicate removal, 512 articles remained. After title–abstract screening, 38 articles were identified for eligibility for full text screening. Final number of studies included in the systematic review after full text screening *n* = 14.

### 2.6. Study Characteristics

An overview of the patient characteristics and study design details is shown in Table 1 and Table 2 respectively. The imaging modality of choice for myocardial perfusion assessment was SPECT (*n* = 5), PET (*n* = 2), MRI (*n* = 3), or ICA (*n* = 4). The vasodilator agent used was dipyridamole (*n* = 3), adenosine triphosphate (ATP) (*n* = 2), adenosine (*n* = 9), or regadenoson (*n* = 3).

### 2.7. Study Quality

Study quality was assessed with a method based on the Quality Assessment of Diagnostic Accuracy Studies (QUADAS) forms. For the purpose of this systematic review on the effects of caffeine on myocardial perfusion measurements, the following study design components were assessed and graded as either low, high, or unclear risk of bias or applicability concern: 1. Patient selection (low: Prospective patients without inappropriate exclusion, high: (Pre-)selection based on imaging results or measurements, unclear: Not specified); 2. Intervention (low: Serum caffeine level was >4 mg/L, high: Serum caffeine levels <4 mg/L, unclear: Not specified); 3. Analysis (low: Analysis was interpreted without knowledge of the intervention, high: Analysis was interpreted without adequate blinding of the intervention status, unclear: Not specified); 4. Time interval between caffeine intervention and analysis (low: >30 min between caffeine intervention and analysis, high: <30 min between caffeine intervention and analysis, unclear: Not specified). The results of the study quality assessment are shown in Table 3. All studies included in this systematic review are at high risk of selection-bias either due to pre-selection of the study population based on imaging results (presence/absence of ischemia, presence of significant stenosis) or due to inclusion of only healthy volunteers.

## 3. Discussion

The competitive nature of caffeine for the adenosine receptor poses a threat to the validity of all myocardial perfusion modalities, irrespective of the vasodilator being used. In the past three decades, several publications have attempted to assess the impact of (recent) caffeine ingestion on the perfusion examinations. This systematic review aims to provide an overview of the available data and discusses the impact of caffeine ingestion on the different perfusion modalities and vasodilator agents.

Currently, the fractional flow reserve (FFR), as measured during invasive coronary angiography (ICA), is regarded as the reference standard for the functional assessment of myocardial perfusion. The guidelines state that FFR measurements should be performed in case of uncertainty regarding the significance of a coronary stenosis and that FFR measurements should be performed in case of intermediate stenosis (40–70%) [18]. Other imaging modalities that have the potential to provide functional information on myocardial pefusion are SPECT, PET, CT, and MRI. A recent meta-analysis focusing on the diagnostic accuracy of these cardiac perfusion imaging modalities showed a superior diagnostic accuracy of PET, MRI, and CT as compared to SPECT perfusion imaging [19]. SPECT imaging suffers from a limited spatial resolution and as a result, subtle differences in myocardial perfusion are more likely to be missed.

### 3.1. SPECT

Three out of five SPECT studies included in this systematic review reported a non-significant effect of recent caffeine ingestion on the functional perfusion measurement [20,21,22]. The studies by Lee et al. and Zoghbi et al. selected patients with ischemia on baseline SPECT and performed a second SPECT after caffeine ingestion [20,21]. They reported no significant effect of caffeine ingestion on MPI. However, both report a relatively low serum concentration of caffeine prior to performing the second MPI, possibly underestimating the effect of caffeine. The study by Reyes et al. selected patients with ischemia on baseline SPECT and performed a second SPECT after caffeine intervention (200 mg orally) with either the standard dosage of 140 µg/kg/min (*n* = 12) or increased dosage of 210 µg/kg/min (*n* = 18) [22]. The reported serum concentration of caffeine in this study was higher in both groups when compared to Lee et al. and Zoghbi et al. A significant effect of caffeine on the functional perfusion measurement in the group with standard adenosine dosage, but no significant effect in the group with the increased adenosine dosage, was detected, suggesting that the effect of caffeine can be overcome by an increased dosage of the vasodilator agent. Smits et al., report a significantly lower redistribution score as measured on dipyridamole-SPECT after intravenous injection of caffeine compared to baseline SPECT [23]. The serum caffeine concentration in this study was relatively high, potentially securing a maximal effect of the caffeine intervention. The placebo-controlled study by Tejani et al. with large sample size showed a significant decrease in the number of ischemic segments by caffeine measured during regadenoson-SPECT as compared to placebo [24]. When considering the study quality assessment, the two studies reporting no significant effects of the caffeine intervention on the perfusion measurement score worse as compared to the studies reporting significant effects, primarily driven by a lower serum concentration of caffeine.

### 3.2. PET

The two PET studies that report on the effects of caffeine on the functional perfusion measurements show a significant reduction in the myocardial flow reserve and myocardial blood flow, all at relatively low serum concentrations of caffeine during either dipyridamole or ATP induced hyperemia [25,26]. However, it should be noted that both studies included healthy individuals, making translation to clinical practice difficult.

### 3.3. MRI

All three studies that report on the effects of caffeine on adenosine MRI indicate a significant effect on the perfusion measurements [17,27,28]. Greulich et al., showed that caffeine one hour before the perfusion measurement at a serum level of 4.6 ± 2.2 mg/L caused a significant decrease in the ischemic burden [27]. In the other two studies, serum caffeine concentration is not reported. However, both studies report a significant effect on the Coronary Sinus Flow Reserve (CsFR) and T1-reactivitity, respectively [17,28]. In the study by our research group, the T1-reactivity appeared unaffected by recent caffeine intake in patients that underwent regadenoson perfusion MRI [28].

### 3.4. ICA

The four studies assessing the effects of recent caffeine intake on the FFR used either ATP or adenosine as the vasodilator agent. The study by Nakayama et al., showed a significantly higher mean FFR value after caffeine ingestion at a “low” (140 µg/kg/min) and “high” (170 µg/kg/min) dose of ATP [29]. Matsumoto et al., also indicate a significant effect of recent caffeine ingestion on the FFR measurement at adenosine dosages of 140 µg/kg/min, 170 µg/kg/min, and 210 µg/kg/min [30]. Mutha et al. and Aqel et al. both report a non-significant effect of intravenous administration of caffeine 5–10 min before the FFR measurement [31,32]. Interestingly, the mean FFR values in the study by Mutha et al. do suggest a significant effect [31]. The lack of significance in this study might be due to the small study population, as they only included ten patients. The study does report that in 2 out of the 10 patients, the FFR value changed from significant (≤0.8) to non-significant (>0.8) after caffeine administration [31]. The study by Aqel et al., shows no significant effect of intravenous caffeine administration at a low serum concentration of caffeine and also in a small study population of only ten patients [32]. Additionally, the time interval between the coffee intervention and the perfusion measurement in the studies by Mutha et al. and Aqel et al. was only several minutes, which is possibly insufficient time for the caffeine to cause a maximal effect. The short time interval between caffeine intervention and the perfusion measurement is also not a good representation of clinical practice.

### 3.5. Contributing Factors

When summarizing the presented data on the potential effects of recent caffeine ingestion on functional perfusion measurements, several study design details appear to have an effect on the outcome. First of all, the different vasodilator agents appear to have a different sensitivity for recent caffeine ingestion, which also seems to be dose dependent. Almost all of the PET, MRI, and ICA studies reporting on the effects of caffeine on adenosine perfusion imaging at the standard dosage of 140 µg/kg/min show a significant effect on the perfusion parameter [17,25,26,27,28,30,31]. Only two ICA studies report non-significant effects [29,30]. As discussed in the previous section, possible explanations for the lack of a significant effect in these studies are the small study population, the timing of caffeine intervention, and the low serum concentrations of caffeine, which does not reflect the clinical setting. Additionally, the SPECT studies with a reasonable time interval between caffeine intervention and the perfusion measurement also indicate a significant effect on the perfusion measurement [23,24]. The SPECT and PET studies reporting on dipyridamole perfusion imaging show a significant effect of recent caffeine ingestion [23,25,26]. The effects of recent caffeine ingestion on regadenoson perfusion imaging remain unclear. Only two papers included in this systematic review report on the possible effects of caffeine on regadenoson, and these papers show contradictory results without a clear indication for the difference [24,28]. Regadenoson is increasingly used as the vasodilator agent of choice for perfusion measurement, and further research should be conducted to better understand the influence of caffeine intake on regadenoson perfusion.

### 3.6. Clinical Relevance

For translation to clinical practice, it is essential to investigate if the effects of caffeine on the perfusion measurements change clinical decision making. Current guidelines of the European Society of Cardiology (ESC) indicate an area of ischemia ≥10% as high risk and a class IB indication for revascularization for both improvement of prognosis and persisting symptoms under Optical Medical Therapy (OMT) [33]. For MRI, this roughly translates to ≥2 segments with new perfusion defects. The SDS score is used in SPECT analysis, with a score of >8 indicating “severe ischemia” [34]. During ICA, a cut-off value of ≤0.80 is used to indicate stenosis with guideline based indication of revascularization [33].

Only a few articles included in this review provide information that can be used to make a statement on the possible clinical relevance. The MRI study by Greulich et al. reports that no conversion of a positive to a negative stress study occurred on a per patient basis, although the mean ischemic burden was significantly reduced by one segment after caffeine administration [27]. However, it must be noted that the study population consisted of a relatively diseased population with a high baseline mean number of ischemic segments (7.9 ± 3.5), meaning that in this specific population, re-classification as a result of caffeine ingestion would only occur if the detected ischemic burden would be reduced with ≥6 segments by caffeine. Especially at the lower ranges of ischemic burden, the reduction of a small amount of segments by caffeine ingestion might result in re-classification. The SPECT study by Reyes et al. shows a re-classification of the SDS of patients from severe to mild-moderate at the standard adenosine dosage of 140 µg/kg/min [22]. With strict adherence to the guidelines, this would mean that these patients would not be referred for further treatment based on their ischemia burden. Both Zoghbi et al. and Lee et al. report the presence of non-significant ischemia at baseline without a change of classification after caffeine administration [21,20]. These results are to be expected, as the general hypothesis is that caffeine administration might potentially lower the amount of detected ischemia and not increase it, making “re-classification” in these studies impossible. The ICA study by Matsumoto et al. indicates a possible clinical relevant effect of caffeine on FFR measurements [30]. As stated previously, the current cut-off value of FFR for indicating relevant myocardial ischemia is ≤0.80. The mean FFR value at baseline in their study population with adenosine dosage of either 140 µg/kg/min, 170 µg/kg/min, or 210 µg/kg/min indicates significant disease with an FFR ≤0.8 (FFR 0.78 ± 0.09 papaverine). After caffeine ingestion, the mean FFR values in the 140 µg/kg/min and 170 µg/kg/min groups change from significant to non-significant >0.8 (FFR after caffeine administration 0.81 ± 0.09), clearly indicating the potential of recent caffeine ingestion to cause re-classification during ICA.

### 3.7. Stress Adequacy

The T1-reactivity can be used as an imaging biomarker for the assessment of stress adequacy during vasodilator perfusion MRI. It is useful to measure stress adequacy either before the perfusion acquisition or retrospectively during image post-processing and evaluation [35]. We believe that reporting the T1-reactivity will aid in the proper interpretation of MRI perfusion images and that the imaging biomarker should be used as a quality check for stress adequacy.

## 4. Conclusions

When considering the studies with high study quality, the available data indicate a significant influence of recent caffeine intake on cardiac perfusion measurements during adenosine and dipyridamole induced hyperemia in SPECT, PET, MRI, and ICA. Recent caffeine ingestion prior to functional perfusion measurements has the potential to affect clinical decision making by re-classification to different risk-categories.

## Implications of Key Findings

Caffeine intake prior to perfusion measurements should be discouraged and in case of recent caffeine intake, rescheduling of the procedure or switching to regadenoson as the vasodilator agent of choice should be considered. During vasodilator perfusion MRI, the T1-reactivity can be used as a biomarker to assess stress adequacy and to indicate patients at risk of false-negative perfusion results.

## Figures and Tables

**Table 1:** Patient characteristics of the studies included in the systematic review. Variables either presented as n, mean ± SD or *n* (%). CAD: coronary artery disease; *N*: number of patients; BMI: Body mass index; MPI: Myocardial perfusion imaging. * Intervention/control: Number of patients with caffeine intervention/number of patients without caffeine intervention.

	*N*	Intervention/Controls *	Study Population	Age	Male	BMI
SPECT						
Smits 1991	8	8/0	Ischemia on baseline MPI	60 ± 7	3(38)	28 ± 4
Zoghbi 2006	30	30/0	Ischemia on baseline MPI	64 ± 9	22(73)	NS
Reyes 2008	30	12/0	Ischemia on baseline MPI	66 ± 6	NS	29 ± 4
		18/0	Ischemia on baseline MPI	64 ± 7	NS	27 ± 3
Lee 2012	30	30/0	Ischemia on baseline MPI	70 ± 8	21(70)	NS
Tejani 2014	207	0/66 70/0 71/0	Ischemia on baseline MPI	68 ± 10.0/65.7 ± 11/69.4 ± 8.2	55(83.3)/58(82.9/51(71.8)	NS
PET						
Böttcher 1995	12	12/0	healthy volunteers	27 ± 6	7(58)	NS
Kubo 2004	10	10/0	healthy volunteers	31 ± 6	10(100)	NS
	10	10/0	healthy volunteers	31 ± 6	10(100)	NS
MRI						
Carlsson 2015	16	16/0	healthy volunteers	41 ± 3	8(50)	NS
Greulich 2017	30	30/0	Ischemia on baseline MPI	68 ± 8	25(83)	NS
van Dijk 2017	98	15/50	suspected of CAD	65 ± 11	46(49)	NS
		9/24	suspected of CAD	65 ± 11	46(49)	NS
ICA						
Matsumoto 2014	42	28/14	Intermediate stenosis	70 ± 8/69 ± 10	21(75)/11(79)	24 ± 3/23 ± 4
	42	28/14	Intermediate stenosis	70 ± 8/69 ± 10	21(75)/11(79)	24 ± 3/23 ± 4
	42	28/14	Intermediate stenosis	70 ± 8/69 ± 10	21(75)/11(79)	24 ± 3/23 ± 4
Mutha 2014	10	10/0	Intermediate stenosis	60 ± 9	8(80)	NS
Aqel 2004	10	10/0	patients with CAD	53 ± 8	10(100)	NS
Nakayama 2018	30	15/15	patients with significant CAD	69 ± 10	25(83)	24 ± 3
	30	15/15	patients with significant CAD	69 ± 10	25(83)	24 ± 3

**Table 2:** Study design details concerning vasodilator agent, caffeine, and main findings. * Timing of caffeine intervention prior to the examination. Continuous variables either reported as mean ± standard deviation or median (interquartile range).

	Vasodilator	Dosage	Caffeine Dosage	Serum Concentration	Timing *	Main Finding	*p*-Value
SPECT							
Smits 1991	Dipyridamole	0.56 mg/kg	4 mg/kg i.v.	9.7 ± 1.3 mg/L	30 min	Redistribution score caffeine 2.0 ± 1.1 vs. 9.0 ± 0.9 baseline	<0.05
Zoghbi 2006	Adenosine	140 µg/kg/min	8 oz cup of coffee	3.1 ± 1.6 mg/L	1 h	SDS caffeine 3.9 ± 2.3 vs. 3.8 ± 1.9 without caffeine	0.8
Reyes 2008	Adenosine	140 µg/kg/min	2 shots espresso	6.2 ± 2.6	1 h	SDS caffeine 4.1 ± 2.1 vs. baseline 12.0 ± 4.4	<0.001
	Adenosine	210 µg/kg/min	2 shots espresso	5.7 ± 2.0	1 h	SDS caffeine 7.8 ± 4.2 vs. baseline 7.7 ± 4.0	0.7
Lee 2012	Adenosine	140 µg/kg/min	one cup of coffee	3.4 mg/L range 0.7–10.4	1 h	mean difference stress percent defect −1.6	0.3
Tejani 2014	Regadenoson	400 µg	placebo, 200 mg or 400 mg caffeine orally	NS	1.5 h	mean difference number of ischemic segments after 200 mg −0.61 ± 1.097, 400 mg −0.62 ± 1.367, placebo −0.12 ± 0.981	<0.001
PET							
Böttcher 1995	Dipyridamole	560 µg/kg	1–2 cups of coffee	range 0–8 mg/L	1–4 h	Flow reserve caffeine 2.3 ± 0.7 vs. 3.4 ± 0.8	<0.001
Kubo 2004	Dipyridamole	560 µg/kg	2–3 cups of coffee	3.3 ± 1.3 mg/L	1.5 h	MFR caffeine 2.25 ± 0.94 vs. baseline 4.11 ± 1.44	<0.005
	ATP	160 µg/kg/min	2–3 cups of coffee	3.1 ± 1.6 mg/L	1.5 h	MFR caffeine 2.44 ± 0.88 vs. baseline 5.15 ± 1.64	<0.005
MRI							
Carlsson 2015	Adenosine	140 µg/kg/min	minimal 6 g instant coffee	NS	12 vs. 24 h	CsFR 12 h 4.31 ± 0.57 vs. 24 h 5.32 ± 0.76	0.03
Greulich 2017	Adenosine	140 µg/kg/min	200 mg orally	4.6 ± 2.2 mg/L	1 h	Ischemic burden 6.9 ± 3.5 caffeine vs. 7.9 ± 3.5 baseline	<.001
van Dijk 2017	Adenosine	140 µg/kg/min	1–2 cups of coffee	NS	<4 h	T1 reactivity caffeine −7.8 ± 5.0 vs. control 4.3 ± 2.8	<0.001
	Regadenoson	400 µg	1–2 cups of coffee	NS	<4 h	T1 reactivity caffeine 4.4 ± 3.2 vs. control 5.4 ± 2.4	0.4
ICA							
Matsumoto 2014	Adenosine	140 µg/kg/min	20 patients 100 or 200 mg orally	2.9[1.8–4.6] mg/L	NS	FFR caffeine 0.81 ± 0.09 vs. 0.78 ± 0.09 papaverine	<0.001
	Adenosine	170 µg/kg/min	20 patients 100 or 200 mg orally	2.9[1.8–4.6] mg/L	NS	FFR caffeine 0.81 ± 0.09 vs. 0.78 ± 0.09 papaverine	<0.01
	Adenosine	210 µg/kg/min	20 patients 100 or 200 mg orally	2.9[1.8–4.6] mg/L	NS	FFR caffeine 0.79 ± 0.09 vs. 0.78 ± 0.09 papaverine	0.01
Mutha 2014	Adenosine	140 µg/kg/min	4 mg/kg i.v.	16.4 ± 5.5 mg/L	7 min	FFR caffeine 0.82 ± 0.11 vs. 0.79 ± 0.07 baseline	0.15
Aqel 2004	Adenosine	30–50 µg bolus i.c.	4 mg/kg i.v.	3.8 ± 1.3 mg/L	5 min	FFR caffeine 0.75 ± 0.14 vs. 0.76 ± 0.13	0.7
Nakayama 2018	ATP	140 µg/kg/min	222 mg orally	7.3 ± 2.0 mg/L	2 min	FFR caffeine 0.78 ± 0.12 vs. FFR papaverine 0.75 ± 0.14	0.002
	ATP	170 µg/kg/min	222 mg orally	7.3 ± 2.0 mg/L	2 h	FFR caffeine 0.77 ± 0.12 vs. FFR papaverine 0.75 ± 0.14	0.007

**Table 3:** Study quality assessment. Red: High, Low: Green, Orange: Unclear risk of either bias (patient selection bias, analysis bias) or applicability concerns (intervention and timing interval possibly not reflection of clinical practice).

	Patient Selection	Intervention	Analysis	Timing Interval
SPECT				
Smits 1991				
Zoghbi 2006				
Reyes 2008				
Lee 2012				
Tejani 2014				
PET				
Böttcher 1995				
Kubo 2004				
CMR				
Carlsson 2015				
Greulich 2017				
van Dijk 2017				
ICA				
Matsumoto 2014				
Mutha 2004				
Aqel 2004				
Nakayama 2018				
